# The benefit of combinations of oximes for the ability of antidotal treatment to counteract sarin-induced brain damage in rats

**DOI:** 10.1186/s40360-018-0227-0

**Published:** 2018-06-28

**Authors:** Filip Caisberger, Jaroslav Pejchal, Jan Misik, Jiri Kassa, Martin Valis, Kamil Kuca

**Affiliations:** 1 Department of Neurology, University Hospital Hradec Kralove, Hradec Kralove, Czech Republic; 2 Department of Toxicology and Military Pharmacy, Faculty of Military Health Sciences, University of Defence, Hradec Kralove, Czech Republic; 3 Department of Chemistry, Faculty of Science, University of Hradec Kralove, Hradec Kralove, Czech Republic; 4 Biomedical Research Center, Uiversity Hospital Hradec Kralove, Hradec Kralove, Czech Republic

**Keywords:** Sarin, HI-6, Trimedoxime, K203, Rats, Histopathology, FluoraJadeB, TUNEL

## Abstract

**Background:**

The aim of our study was to compare the ability of two combinations of oximes (HI-6 + trimedoxime and HI-6 + K203) with atropine to counteract acute sarin-induced brain damage with the efficacy of antidotal treatment involving single oxime (HI-6) and atropin using in vivo methods.

**Methods:**

Brain damage and neuroprotective effects of antidotal treatment were evaluated in rats poisoned with sarin at a sublethal dose (108 μg/kg i.m.; 90% LD_50_) using histopathological, Fluoro-Jade B and Terminal deoxynucleotidyl transferase dUTP nick end labeling (TUNEL) analysis 24 h after sarin administration.

**Results:**

Both combinations of oximes reduce the number of rats that died before the end of experiment compared to non-treated sarin poisoning and sarin poisoning treated with HI-6 and atropine. In the case of treatment of sarin poisoning with HI-6 in combination with K203, all rats survived till the end of experiment. HI-6 with atropine was able to reduce sarin-induced brain damage, however, both combinations were slightly more effective.

**Conclusions:**

The oxime HI-6 in combination with K203 and atropine seems to be the most effective. Thus, both tested oxime combinations bring a small benefit in elimination of acute sarin-induced brain damage compared to single oxime antidotal therapy.

## Background

Nerve agents are highly toxic organophosphorus compounds representing potential threats to both military and civilian population. These chemical warfare agents were used in the Iraq-Iran (1980s), in the terrorist attack on the Tokyo subway (1995) and more recently in Syria in the suburbs of Damascus resulting in massive casualties [32, 36]. The basic mechanism of nerve agents´ toxicity lies in irreversible inhibition of acetylcholinesterase (AChE, EC 3.1.1.7) leading to the accumulation of the neurotransmitter acetylcholine at cholinergic synapses and neuromuscular junctions which subsequently results in the cholinergic crisis involving multiple organs. In the central nervous system, nerve agents may induce progressive irreversible damage [4]. The brain damage is associated with the dysfunction of irreversibly inhibited AChE and neuronal excitotoxicity and it seems to be largely responsible for persistent profound neuropsychiatric and neurological impairments in the victims of nerve agent exposure [4, 6].

The standard treatment of nerve agent poisoning usually consists of three types of antidotes – anticholinergic agents, oximes and anticonvulsive drugs. Anticholinergic drugs such as atropine sulphate antagonize the muscarinic overstimulation, anticonvulsants such as diazepam are indicated to prevent centrally mediated seizures and secondary brain damage and the oximes disrupt the covalent bond between nerve agent and AChE and restore the physiological function of this enzyme [8]. Among oximes, monopyridinium oxime pralidoxime and bispyridinium oximes obidoxime, trimedoxime and HI-6 should be mentioned [39]. However, there is no potent broad-spectrum oxime able to sufficiently reactivate AChE inhibited by all nerve agents regardless of their chemical structure. HI-6, for instance, is considered to be a relatively strong reactivator of sarin, soman and cyclosarin inhibited AChE but its ability to reactivate AChE inhibited by tabun and less toxic organophosphorus pesticides is of low to moderate values [7, 17, 21, 26, 39, 41]. Trimedoxime or newly synthesized oxime K203, on the other hand, seem to be effective against tabun and pesticides [22, 24]. Since the effort to develop a potent broad-spectrum reactivator has failed so far, another way how to overcome this issue and possibly to increase the reactivating efficacy of antidotes is to combine the oximes in the antidotal treatment [19, 41].

The main purpose of this study was to compare the neuroprotective efficacy of the oxime HI-6 with two oxime mixtures containing HI-6 and trimedoxime or the oxime K-203 in combination with atropine in sarin-poisoned rats. The neuroprotective potential was evaluated using histopathological examination with the help of Fluoro-Jade B fluorochrome used in neuroscience to label degenerating neurons and terminal deoxynucleotidyl transferase dUTP nick end labeling (TUNEL) assay detecting DNA fragmentation during apoptosis [25, 37].

## Methods

### Animals

All experiments in this study were approved by the Ethics Committee of the Faculty of Military Health Sciences in Hradec Kralove (Czech Republic) and were conducted in accordance with the Animal Protection Law and Animal Protection Regulations.

Wistar rats (6-week-old, 200–230 g; VELAZ, Unetice, Czech Republic) were kept in an accredited animal facility (22 ± 2 °C, 50 ± 10% relative humidity, 12-h day-night cycle) and allowed access to standard food (Velaz) and tap water ad libitum. The rats were acclimatized for 14 days before starting the experiments. For experiments, they were randomly divided into groups of 8 animals.

### Chemicals

Sarin was obtained from Military Technical Institute (Brno, Czech Republic). Its purity (98%) was assayed by acidimetric titration. Trimedoxime (1,1′-propane-1,3-diylbis{4-[(*E*)-(hydroxyimino)methyl]pyridinium} dibromide), HI-6 (1-[[[4(aminocarbonyl)-pyridinio] methoxy]methyl]-2(hydroxyimino)pyridinium dichloride) and the oxime K203 [(E)-1-(4-carbamo-ylpyridinium)-4-(4-hydroxyiminomethylpyridinium)-but-2-ene dibromide] were synthesized earlier at the Department of Toxicology (Faculty of Military Health Sciences, Hradec Kralove, Czech Republic). Their purities (97.5%) were analyzed using high-performance liquid chromatography technique [11]. All other drugs and chemicals of analytical grade were obtained commercially and used without further purification. All substances were administered intramuscularly at a volume of 1 ml/kg of body weight.

### In vivo experiments

Animals were divided into 5 groups (8 rats each): 1) a control group (administered with saline), 2) sarin poisoned group (108 μg/kg – 90% LD_50_), 3) sarin poisoned group treated with atropine sulphate (21 mg/kg) and HI-6 (39 mg/kg), 4) sarin poisoned group treated with atropine sulphate, HI-6 and trimedoxime (7.5 mg/kg), and 5) sarin poisoned group treated with atropine sulphate, HI-6 and K203 (16.3 mg/kg). In order to develop a broad-spectrum mixture, the combinations were selected based on HI-6 being a relatively strong reactivator of sarin, soman and cyclosarin-inhibited AChE, while trimedoxime or K203 are effective against tabun and pesticides. The oximes were administered at equitoxic doses corresponding to 5% of their LD_50_ values. These doses are sufficiently safe. The antidotes were administered 1 min after sarin challenge. Surviving rats were anesthetized by ether vapor 24 h after intoxication and euthanized by decapitation. Their brains were rapidly removed and fixed with a 10% neutral buffered formalin (Chemapol, Prague, Czech Republic). Samples were subsequently embedded into paraffin (Paramix, Holice, Czech Republic) and 6 μm thick coronary brain sections were cut (Microtome model SM2000 R, Leica) at level between 3 mm and 4.2 mm from bregma according to Paxinos stereotactic brain atlas [33]. Three sections for every staining method were evaluated by 2 experts. In a section, damage was scored in 6 regions, including amygdaloid body, cortex, hippocampus, hypothalamus, piriform cortex and thalamus with relevance to sarin-induced neuropathology [13].

### Hematoxylin-eosin staining and histopathology evaluation

Selected brain sections were fixed in 10% buffered formalin (Kulich, Hradec Kralove, Czech Republic), processed through conventional histological techniques, and stained with hematoxylin and eosin (both Merck, Kenilworth, NJ, USA). The histological changes were scored using a BX-51 microscope (Olympus, Tokyo, Japan) and following semi-quantitative criteria published previously [20]:

(0) no pathology,
(1) mild damage: 1–2 neurons with nucleus margination, chromatolysis and/or vacuolar degeneration,
(2) moderate damage: ≥ 3 neurons with changes described in (1) and/or ≥ 1 shrunken eosinophilic neurons in 1 nucleus/subregion,
(3) severe damage: focal damage or multiple changes described in (2) present in ≥2 nuclei/subregions and/or hemorrhage
(4) very severe damage: diffuse damage of the region and/or multiple hemorrhages

### Fluoro-jade B staining and evaluation

For Fluoro-Jade B staining, paraffin sections were dewaxed and rehydrated through xylene and an alcohol series. Afterwards, the sections were immersed in 0.06% potassium permanganate (Sigma-Aldrich, St. Louis, MO, USA) for 10 min, rinsed in distilled water for 2 min and dyed by 0.0002% solution of the Fluoro-Jade B (Merck) for 20 min in dark. Washed (3 distilled water washes, 1 min each) and dried slides were mounted with DPX (Sigma-Aldrich) in dark. Fluorescent cells were evaluated using a BX-51 microscope (Olympus) with green excitation fluorescence filter and following semi-quantitative criteria published previously [20]:

(0) no fluorescent cells,
(1) 1–2 fluorescent cells in 1 nucleus/subregion,
(2) ≥ 3 in 1 nucleus/subregion or 1–2 fluorescent cells in ≥2 nuclei/subregions,
(3) multiple fluorescent cells in ≥2 nuclei/subregions and/or hemorrhage,
(4) diffuse fluorescent positivity and/or multiple hemorrhages

### TUNEL immunostaining and evaluation

TUNEL staining was carried out to identify the apoptotic cells according to the manufacturer’s instructions using in situ cell death detection kit (Roche, Mannheim, Germany) as published previously [34]. TUNEL positive cells were evaluated using a BX-51 microscope (Olympus) and following semi-quantitative criteria:

(0) 0–1 TUNEL positive cells in 1 nucleus/subregion
(1) ≥ 2 TUNEL positive cells in 1 nucleus/subregion
(2) multiple TUNEL positive cells in ≥2 nuclei/subregions
(3) diffuse TUNEL positivity

Due to verified false positivity of TUNEL assay and a very slight positivity (1 cell) in all control samples, only 3 levels of damage were recognized compared to the histopathological and Fluoro-Jade B analysis.

### Individual animal brain damage score

Finally, individual animal brain damage scores were calculated representing a sum of histopathological, Fluoro-Jade B and TUNEL scores from all tested brain subregions in one animal.

### Data analysis

We used non-parametric Kruskal-Wallis rank sum test with multiple pairwise comparisons to evaluate statistical differences between all groups within particular brain nucleus/subregion, which were considered significant when *p* ≤ 0.05. All analyses were performed in IBM SPSS Statistics version 22.0 software (IBM Corp., Armonk, NY, USA).

## Results

In the case of sarin poisoning without antidotal treatment, three sarin-poisoned rats did not survive until the end of experiment. Antidotal treatment eliminated or at least reduced the mortality of sarin-poisoned rats. Two rats did not survived till the end of experiment if HI-6 in combination with atropine was used for the antidotal treatment and one rat did not survived till the end of experiment if HI-6 and trimedoxime in combination with atropine were used for the antidotal treatment of sarin poisoning. Only HI-6 and K203 in combination with atropine were able to eliminate sarin-induced death of poisoned animals (Table 1).

**Table 1:** Survival of rats following exposure to sarin

group	survival in the time of sample collection (survived/total)	time of death (min)
control	8/8	
GB	5/8	28, 42, 63
GB + A + H	6/8	33, 125
GB + A + H + T	7/8	59
GB + A + H + K	8/8	

### Histopathological findings

**Fig. 1:**
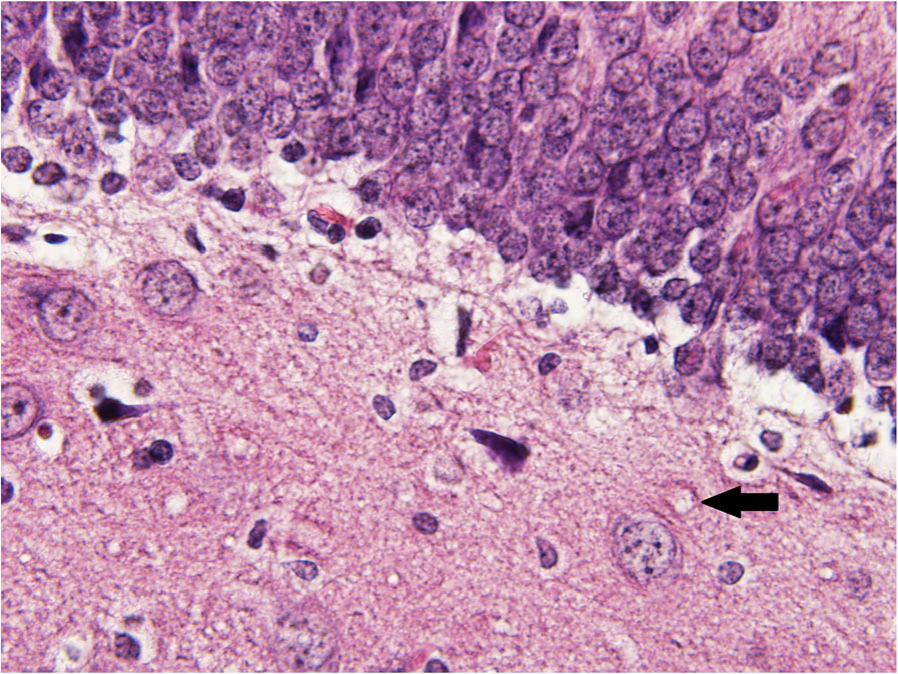
Sample of dentate gyrus from control group (hematoxylin-eosin, 600fold original magnification). The microphotograph displays a boundary between granular (upper) and polymorphic (lower) layer. The arrow indicates an example of a neuronal vacuole surrounded with condesated cytoplasm

**Fig. 2:**
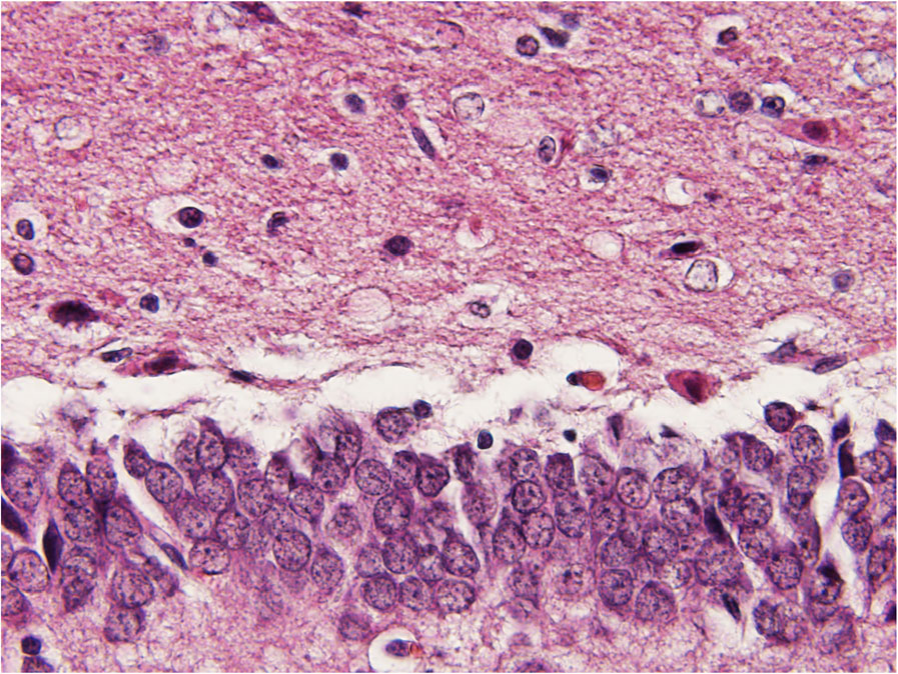
Sample of dentate gyrus from sarin-poisoned rat receiving no treatement (hematoxylin-eosin, 600fold original magnification). The microphotograph displays a boundary between polymorphic (upper) and granular (lower) layer. Sarin-induced damage dominates in polymorphic layer and includes multiple neuronal vacuoles surrounded with condensated chromatin and red cells with condensated nuclei (acute eosinophilic necrosis)

The most extensive alterations were observed in sarin-poisoned animals receiving no antidotal treatment. Compared to the control group, significantly increased histopathological damage scores were measured in cortex, hypothalamus and piriform cortex (*p* = 0.023, 0.001 and 0.020, respectively). In affected animals, cortex and piriform cortex displayed pseudolaminar necrosis in 1 and 3 cases, respectively. Hemorrhage was present in one case in hypothalamus. Single cell damage characterized by multifocal degenerative/necrotic changes accompanied with oedema was observed in the remaining positively scored rats. Especially, basolateral nucleus of amygdaloid body, the third and fifth layer of cortex (pyramidal cells), neurons in CA1 (> dentate gyrus > CA3 > CA2) zone of hippocampus (Figs. 1 and 2), supramammilary hypothalamic nuclei, the third layer of piriform cortex, and medial and dorsolateral thalamic nuclei were markedly impaired.

**Fig. 3:**
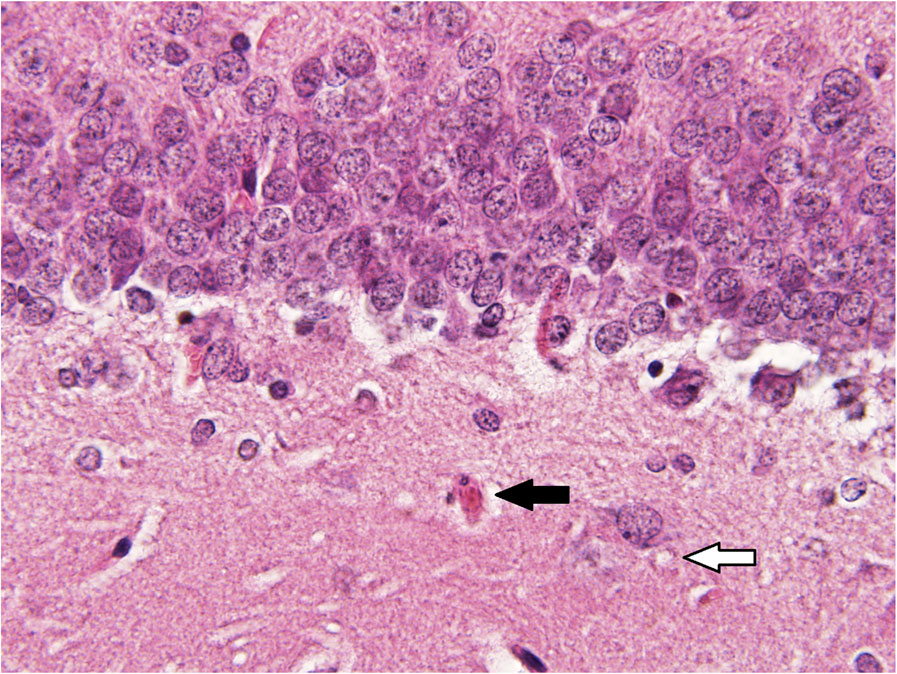
Sample of dentate gyrus from sarin-poisoned rat treated with HI-6 and trimedoxime (hematoxylin-eosin, 600fold original magnification). The microphotograph displays a boundary between polymorphic (upper) and granular (lower) layer. The treatment significantly reduced the pathological finings in this animal, nevertheless, a red cell with chromatolysis (black arrow) and several neuronal vacuoles (white arrow indicates an example) could be observed

Administration of antidotal treatment reduced the extent of damage. Any diffuse changes and/or hemorrhage in all groups treated with antidotes was not observed. However, significantly decreased histopathological scores were found in cortex (*p* = 0.009) of animals administered with atropine and HI-6 and in cortex, hippocampus (Fig. 3), hypothalamus, and piriform cortex (*p* = 0.023, 0.010, 0.009, and 0.020, respectively) in rats treated with atropine, HI-6 and K203 (Table 2).

**Table 2:** Score of sarin-induced histopathological damage in rat brain and its modulation by different therapeutic approaches

	AMG					CTX					HIPP					HYPOTH					PIRI					TH				
score scale	0	1	2	3	4	0	1	2	3	4	0	1	2	3	4	0	1	2	3	4	0	1	2	3	4	0	1	2	3	4
control	6	1	1	0	0	7	1	0	0	0	5	3	0	0	0	8	0	0	0	0	8	0	0	0	0	6	2	0	0	0
GB	2	0	0	3	0	**1**	**1**	**1**	**1**	**1**	1	1	1	0	2	**0**	**0**	**2**	**3**	**0**	**2**	**0**	**0**	**0**	**3**	1	0	1	3	0
GB + A + H	4	1	1	0	0	***6***	***0***	***0***	***0***	***0***	2	4	0	0	0	3	1	2	0	0	5	1	0	0	0	4	2	0	0	0
GB + A + H + T	5	0	2	0	0	5	2	0	0	0	3	3	0	1	0	4	1	2	0	0	6	0	1	0	0	5	1	1	0	0
GB + A + H + K	6	2	0	0	0	***7***	***1***	***0***	***0***	***0***	***8***	***0***	***0***	***0***	***0***	***6***	***1***	***1***	***0***	***0***	***8***	***0***	***0***	***0***	***0***	5	0	3	0	0

Each number represents a number of animals suffering from particular damage score in the group

Bold: statistically significant compared to control group (p ≤ 0.05)

Bold Italic: statistically significant compared to sarin-poisoned group (*p* ≤ 0.05)

*Abbreviations: A* atropine, *AMG* amygdaloid body, *CTX* cortex, *GB* sarin, *H* oxime HI-6, *HIPP* hippocampus, *HYPOTH* hypothalamus, *K* oxime K203, *PIRI* piriform cortex, *T* trimedoxime, *TH* thalamus

### Fluoro-jade B

**Fig. 4:**
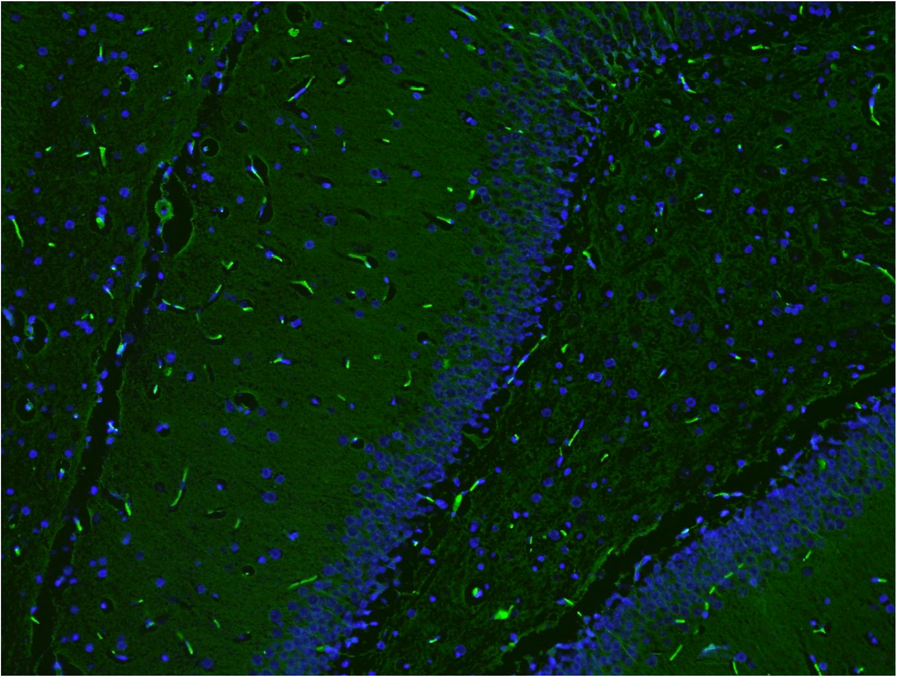
Sample of dentate gyrus from control group (Fluoro-Jade B and DAPI, 100fold original magnification). Autofluorescence of normal red blood cells in capillaries could be seen in the picture. No pathology was detected

**Fig. 5:**
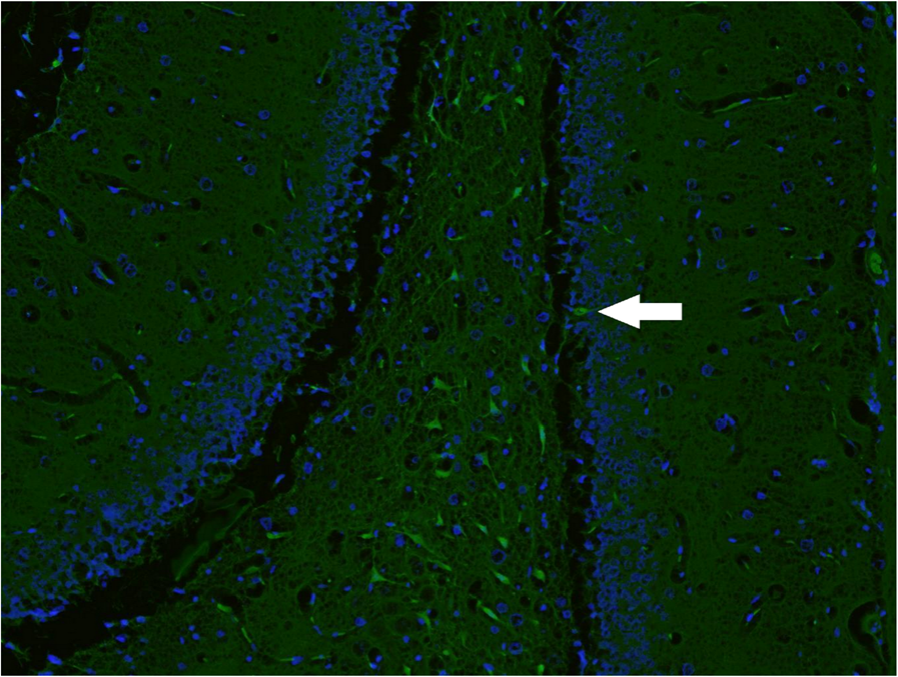
Sample of dentate gyrus from sarin-poisoned rat receiving no treatement (Fluoro-Jade B and DAPI, 100fold original magnification). Together with autofluorescence of normal red blood cells in capillaries, fluorescence of piramidal and polymorphic cells with dendritic and axonal projections (necrotic neurons) in polymorphic layer could be found. The arrow indicates a necrotic neurone in the granular layer

Significantly increased Fluoro-Jade B positivity in amygdaloid body, cortex, hypothalamus, piriform cortex, and thalamus (*p* = 0.029, 0.032, < 0.001, 0.032, and 0.029, respectively) was found in non-treated, sarin-poisoned rats. Diffuse fluorescent patterns were present in 3 of 5 surviving rats. In all six regions, maximal Fluoro-Jade B positivity correlated with histopathological findings (Figs. 4 and 5).

**Fig. 6:**
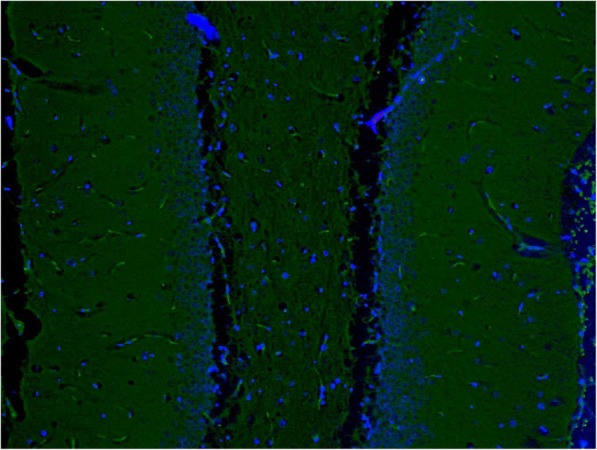
Sample of dentate gyrus from sarin-poisoned rat treated with HI-6 and K203 (Fluoro-Jade B and DAPI, 100fold original magnification). Only autofluorescence of normal red blood cells in capillaries could be seen in the picture. No pathology was detected

The antidotal treatment markedly reduced brain degeneration marked by Fluoro-Jade B. We found significantly decreased Fluoro-Jade B positivity in hippocampus (*p* = 0.017) of atropine and HI-6 treated rats and in amygdaloid body and hippocampus (*p* = 0.038 and 0.026, respectively) of animals administered with atropine, HI-6 and trimedoxime. No hemorrhages were observed in both groups. In atropine, HI-6 and K203 treated group, Fluoro-Jade B fluorescence significantly decreased in cortex, hippocampus (Fig. 6), piriform cortex, and thalamus (*p* = 0.032, < 0.001, 0.032, and 0.029, respectively). On the other hand, a small hemorrhage in the hypothalamic region was found. However, Fluoro-Jade B positivity in this area was scored as 1 (Table 3).

**Table 3:** Score of sarin-induced degeneration (Fluoro-Jade B positivity) in rat brain and its modulation by different therapeutic approaches

	AMG					CTX					HIPP					HYPOTH					PIRI					TH				
score scale	0	1	2	3	4	0	1	2	3	4	0	1	2	3	4	0	1	2	3	4	0	1	2	3	4	0	1	2	3	4
control	8	0	0	0	0	8	0	0	0	0	8	0	0	0	0	8	0	0	0	0	8	0	0	0	0	8	0	0	0	0
GB	**2**	**0**	**0**	**1**	**2**	**2**	**0**	**0**	**2**	**1**	**0**	**2**	**0**	**1**	**2**	**2**	**0**	**0**	**3**	**0**	**2**	**0**	**0**	**0**	**3**	**2**	**0**	**0**	**2**	**1**
GB + A + H	5	0	0	0	1	5	0	0	0	1	***5***	***0***	***0***	***0***	***1***	5	0	0	1	0	4	1	0	1	0	5	0	0	1	0
GB + A + H + T	***7***	***0***	***0***	***0***	***0***	6	0	1	0	0	***5***	***2***	***0***	***0***	***0***	6	0	0	1	0	***6***	***1***	***0***	***0***	***0***	6	0	0	1	0
GB + A + H + K	7	1	0	0	0	***8***	***0***	***0***	***0***	***0***	***8***	***0***	***0***	***0***	***0***	7	0	0	1	0	***8***	***0***	***0***	***0***	***0***	***8***	***0***	***0***	***0***	***0***

Each number represents a number of animals suffering from particular damage score in the group

Bold: statistically significant compared to control group (p ≤ 0.05)

Bold Italic: statistically significant compared to sarin-poisoned group (*p* ≤ 0.05)

*Abbreviations*: *A* atropine, *AMG* amygdaloid body, *CTX* cortex, *GB* sarin, *H* oxime HI-6, *HIPP* hippocampus, *HYPOTH* hypothalamus, *K* oxime K203, *PIRI* piriform cortex, *T* trimedoxime, *TH* thalamus

### TUNEL

**Fig. 7:**
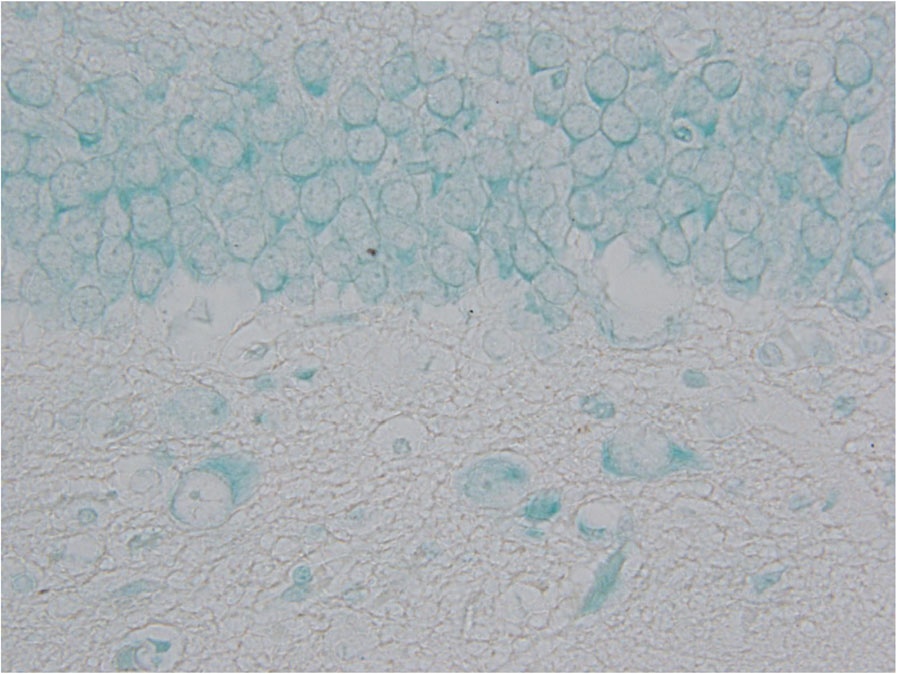
Sample of dentate gyrus from control group (TUNEL assay counterstained with methyl green, 600fold original magnification). The microphotograph displays a boundary between granular (upper) and polymorphic (lower) layer with no TUNEL positivity

**Fig. 8:**
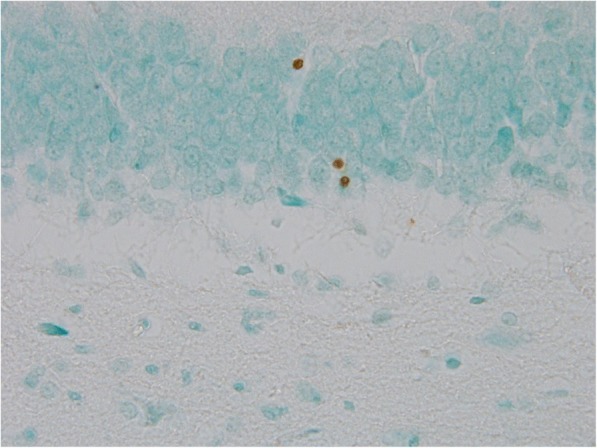
Sample of dentate gyrus from sarin-poisoned animal receiving no treatement (TUNEL assay counterstained with methyl green, 600fold original magnification). The microphotograph displays a boundary between granular (upper) and polymorphic (lower) layer. Three TUNEL positive cells were detected in the granular layer

TUNEL positivity was significantly increased in amygdaloid body, hippocampus and piriform cortex (*p* = 0.012, 0.003 and 0.041, respectively) of sarin-poisoned animals without antidotal treatment. The distribution of TUNEL staining pattern corresponds to the histopathological and Fluoro-Jade B findings except for hippocampal dentate gyrus (Figs. 7 and 8).

**Fig. 9:**
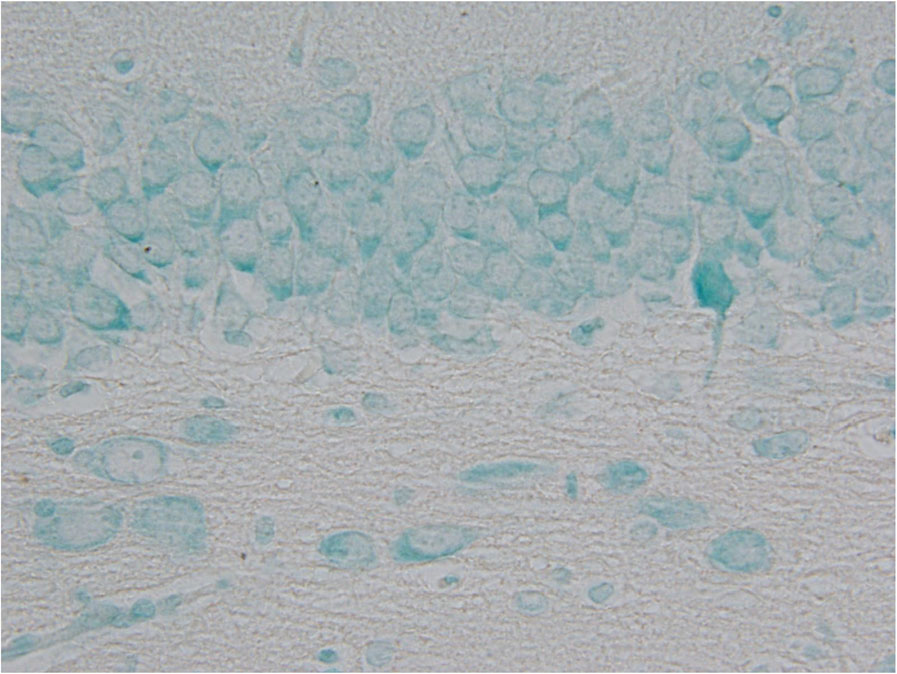
Sample of dentate gyrus from sarin-poisoned rat treated with HI-6 and K203 (TUNEL assay counterstained with methyl green, 600fold original magnification). The microphotograph displays a boundary between granular (upper) and polymorphic (lower) layer with no TUNEL positivity

Atropine and HI-6 treatment did not significantly influence the outcome of sarin intoxication. In atropine, HI-6 and trimedoxime treated group, a decreased amount of TUNEL positive cells was only observed in amygdaloid body (*p* = 0.016). The combination of atropine, HI-6 and K203 decreased positivity in amygdaloid body, hippocampus (Fig. 9) and piriform cortex (*p* = 0.012, 0.003 and 0.041, respectively) (Table 4).

**Table 4:** Score of sarin-induced apoptotic activity (TUNEL positivity) in rat brain and its modulation by different therapeutic approaches

	AMG				CTX				HIPP				HYPOTH				PIRI				TH			
score scale	0	1	2	3	0	1	2	3	0	1	2	3	0	1	2	3	0	1	2	3	0	1	2	3
control	8	0	0	0	8	0	0	0	8	0	0	0	8	0	0	0	8	0	0	0	8	0	0	0
GB	**2**	**2**	**1**	**0**	2	2	1	0	**0**	**0**	**5**	**0**	3	1	1	0	**2**	**1**	**2**	**0**	3	0	2	0
GB + A + H	5	1	0	0	4	1	1	0	2	0	4	0	5	0	1	0	4	2	0	0	2	2	2	0
GB + A + H + T	***7***	***0***	***0***	***0***	6	1	0	0	4	0	3	0	5	0	2	0	6	1	0	0	6	0	1	0
GB + A + H + K	***8***	***0***	***0***	***0***	8	0	0	0	***8***	***0***	***0***	***0***	8	0	0	0	***8***	***0***	***0***	***0***	7	1	0	0

Each number represents a number of animals suffering from particular damage score in the group

Bold: statistically significant compared to control group (p ≤ 0.05)

Bold Italic: statistically significant compared to sarin-poisoned group (p ≤ 0.05)

*Abbreviations*: *A* atropine, *AMG* amygdaloid body, *CTX* cortex, *GB* sarin, *H* oxime HI-6, *HIPP* hippocampus, *HYPOTH* hypothalamus, *K* oxime K203, *PIRI* piriform cortex, *T* trimedoxime, *TH* thalamus

### Individual animal brain damage score

Compared to the control group, a significantly increased individual animal brain damage scores in sarin-poisoned group without antidotal treatment was found (*p* = < 0.001). Sarin-induced damage was significantly reduced only in atropine, HI-6 and K203 treated rats (*p* = 0.019) (Table 5).

**Table 5:** Individual animal brain damage score

	animal No.							
	1	2	3	4	5	6	7	8
control	0	0	0	1	1	2	2	3
GB	**5**	**10**	**47**	**48**	**51**			
GB + A + H	0	3	5	6	7	36		
GB + A + H + T	0	3	3	4	4	5	28	
GB + A + H + K	***0***	***1***	***1***	***3***	***3***	***3***	***3***	***3***

Each number represents a number of animals suffering from particular damage score in the group

Bold: statistically significant compared to control group (p ≤ 0.05)

Bold Italic: statistically significant compared to sarin-poisoned group (*p* ≤ 0.05)

*Abbreviations*: *A* atropine, *GB* sarin, *H* oxime HI-6, *K* oxime K203, *T* trimedoxime

## Discussion

In this study, the neuroprotective effects of single oxime (HI-6) or two oxime mixtures (HI-6 and trimedoxime or oxime K203) in combination with atropine in sarin-poisoned rats was evaluated. The model used in this study follows our previous study, in which the benefit of oxime mixtures for the neuroprotective efficacy of antidotal treatment of nerve agent poisoning was demonstrated using a functional observatory battery [19]. Herein, we focus on structural brain changes using three different approaches, including histopathological, Fluoro-Jade B and TUNEL analysis.

Based on our results, the established scoring system resulted in positivity of histopathological method in 5 (of 8) control animals due to the presence of cytoplasmatic vacuoles. The clinical significance of vacuolation is not known. Neuronal vacuoles can appear spontaneously, especially in aging animals, without having any obvious clinical relevance [23, 40].

After sarin poisoning, a widespread brain damage was observed. The alterations were particularly profound in cholinoceptive subregions and/or subregions sensitive to hypoxic/ischemic stress [3, 27, 29, 35]. This observation is in accordance with generally accepted mechanism of nerve agent-induced acute brain injury and with previously published findings [6, 13, 28]. The distribution of histopathological changes correlated with Fluoro-Jade B and TUNEL staining pattern with one exception. In gyrus dentatus (hippocampus), histopathological findings and Fluoro-Jade B fluorescence showed the highest positivity in the polymorphic layer, whereas TUNEL positivity prevailed in the granular layer. Although both layers display different sensitivity to various stress stimuli [15, 38], the reason of this discrepancy remains uncertain. An explanation may lie in the limitations of TUNEL method. The method is used to detect DNA fragmentation in apoptotic and necrotic cells [2]. On the other hand, DNA fragmentation occurs also in S phase of cell cycle and the method was reported to produce false positivity in proliferating tissues [9]. Since the subgranular zone of dentate gyrus is considered to be one of the two main neurogenic zones in the adult brain [5], TUNEL positivity observed in this subregion could possibly represent the damage as well as its regeneration.

Antidotal treatment plays a pivotal role in acute phase of nerve agents´ toxicity [6]. In our model, we utilized bispyridinium oximes. Although the bispyridinium oximes poorly penetrate across the blood-brain barrier, they are able to reactivate sarin-inhibited AChE not only at the peripheral compartment but also in the brain when they are administered at equitoxic doses corresponding to 5% of their LD_50_ values at 1 min after the administration of sarin [14, 18, 19]. According to our results, all three therapeutic regimes mitigated the extent of sarin-induced brain injury. Even though we did not find any statistically significant differences among groups treated with oximes, the neuroprotective efficacy of oxime mixtures was slightly higher compared to the group treated with a single oxime. Particularly, the combination of HI-6 and K203 appears to be the most effective to protect experimental animals from acute sarin-induced neuropathological changes in surviving animals. This conclusion corresponds to the previously published results demonstrating the benefit of combinations of oximes for the reactivating and therapeutic efficacy of antidotal treatment of sarin poisoning in rats and mice [18]. Based on the described results, both trimedoxime and K203 do not interfere with HI-6 bioavailability but rather support its action. Therefore, combining the oximes in the antidotal treatment could be a promising step towards a broad-spectrum antidotal treatment of acute nerve agent-exposure regardless of the chemical structure of nerve agent. Such approach may also help to overcome similar issue regarding organophosphorus pesticides. HI-6 has only limited reactivation efficacy towards chlorpyrifos, dichlorvos, methamidophos, and paraoxon [1, 12, 30, 31]. Its combination with trimedoxime or K203 might not only improve these results but may also broaden the spectrum of reactivation towards leptophos-oxon and possibly other pesticides [12]. On the other hand, even in HI-6 and K203 group, animals displayed signs of neuronal damage, which tends to progress overtime [13]. Limited therapeutic efficacy is probably related to low blood-brain barrier (BBB) penetration of bispyridinium oximes [10, 16]. Thus, the development and usage of oxime mixtures with better BBB penetration may further increase their ability to counteract the acute toxicity of organophosphorus compounds.

## Conclusion

Our results show that neuroprotective potency of both oxime combinations (HI-6 with trimedoxime and HI-6 with K203) is slightly higher than using HI-6 alone in sarin poisoned model. Particularly, the oxime HI-6 in combination with K203 seems to be the most effective. Using oxime combinations seems to be an effective approach to develop a potent broad-spectrum reactivator.

## Abbreviations

AChE: Acetylcholinesterase
EC: Enzyme commission number
i.m.: Intramuscularly
LD_50_: Median lethal dose
TUNEL: Terminal deoxynucleotidyl transferase dUTP nick end labeling
